# End-tidal carbon dioxide levels during resuscitation and carbon dioxide levels in the immediate neonatal period and intraventricular haemorrhage

**DOI:** 10.1007/s00431-019-03543-0

**Published:** 2019-12-17

**Authors:** Kentaro Tamura, Emma E Williams, Theodore Dassios, Anoop Pahuja, Katie A Hunt, Vadivelam Murthy, Prashanth Bhat, Ravindra Bhat, Anthony Milner, Anne Greenough

**Affiliations:** 1 Women and Children’s Health, School of Life Course Sciences, Faculty of Life Sciences and Medicine, King’s College London, London, UK; 2 Division of Neonatology, Maternal and Perinatal Centre, Toyama University Hospital, Toyama, Japan; 3 Neonatal Intensive Care Centre, King’s College Hospital NHS Foundation Trust, London, UK; 4 The Asthma UK Centre in Allergic Mechanisms of Asthma, Kings College London, London, UK; 5 NIHR Biomedical Research Centre at Guy’s and St Thomas’ NHS Foundation Trust and King’s College, London, UK

**Keywords:** Carbon dioxide, End-tidal carbon dioxide, Intraventricular haemorrhage, Prematurity, Resuscitation

## Abstract

Abnormal levels of end-tidal carbon dioxide (EtCO_2_) during resuscitation in the delivery suite are associated with intraventricular haemorrhage (IVH) development. Our aim was to determine whether carbon dioxide (CO_2_) levels in the first 3 days after birth reflected abnormal EtCO_2_ levels in the delivery suite, and hence, a prolonged rather than an early insult resulted in IVH. In addition, we determined if greater EtCO_2_level fluctuations during resuscitation occurred in infants who developed IVH. EtCO_2_ levels during delivery suite resuscitation and CO_2_ levels on the neonatal unit were evaluated in 58 infants (median gestational age 27.3 weeks). Delta EtCO_2_ was the difference between the highest and lowest level of EtCO_2_. Thirteen infants developed a grade 3–4 IVH (severe group). There were no significant differences in CO_2_ levels between those who did and did not develop an IVH (or severe IVH) on the NICU. The delta EtCO_2_ during resuscitation differed between infants with any IVH (6.2 (5.4–7.5) kPa) or no IVH (3.8 (2.7–4.3) kPA) (*p* < 0.001) after adjusting for differences in gestational age. Delta EtCO_2_ levels gave an area under the ROC curve of 0.940 for prediction of IVH.

*Conclusion*: The results emphasize the importance of monitoring EtCO_2_ levels in the delivery suite.

**Table:** 

**What is Known:** • *Abnormal levels of carbon dioxide (CO*_*2*_*) in the first few days after birth and abnormal end-tidal CO*_*2*_ *levels (EtCO*_*2*_*) levels during resuscitation are associated in preterm infants with the risk of developing intraventricular haemorrhage (IVH).*	
**What is New:** • *There were no significant differences in NICU CO*_*2*_ *levels between those who developed an IVH or no IVH.* • *There was a poor correlation between delivery suite ETCO*_*2*_ *levels and NICU CO*_*2*_ *levels.* • *Large fluctuations in EtCO*_*2*_ *during resuscitation in the delivery suite were highly predictive of IVH development in preterm infants.*

**What is Known:**

• *Abnormal levels of carbon dioxide (CO*_*2*_*) in the first few days after birth and abnormal end-tidal CO*_*2*_ *levels (EtCO*_*2*_*) levels during resuscitation are associated in preterm infants with the risk of developing intraventricular haemorrhage (IVH).*

**What is New:**

• *There were no significant differences in NICU CO*_*2*_ *levels between those who developed an IVH or no IVH.*

• *There was a poor correlation between delivery suite ETCO*_*2*_ *levels and NICU CO*_*2*_ *levels.*

• *Large fluctuations in EtCO*_*2*_ *during resuscitation in the delivery suite were highly predictive of IVH development in preterm infants.*

## Introduction

Improvements in neonatal intensive care have resulted in decreased mortality rates of preterm infants. The development of intraventricular haemorrhage (IVH), however, can result in long-term adverse outcomes. Several studies of preterm infants have demonstrated that abnormal levels of carbon dioxide (CO_2_) within the first few days post-delivery are associated with the development of IVH [1, 2]. We recently reported that preterm infants who developed an IVH had higher end-tidal CO_2_ (EtCO_2_) levels in the delivery suite during resuscitation [3]. It has not, however, been determined whether abnormal EtCO_2_ levels during resuscitation of preterm infants translate into abnormal CO_2_ levels over the subsequent 72 h after birth, and hence, whether it is a prolonged rather than an early insult which results in the development of IVH. Our aim then was to study EtCO_2_ levels in the delivery suite during resuscitation of preterm infants and their relationship to the subsequent CO_2_ levels within the first 3 days after birth and the development of IVH.

In one study of very low birth weight infants, not only high but also low partial pressures of carbon dioxide (pCO_2_) were associated with severe IVH development, as was a greater degree of fluctuation in pCO_2_ levels within the first 4 days after birth [2]. In term infants, undergoing therapeutic hypothermia for hypoxic ischaemic encephalopathy, adverse neurodevelopmental outcomes were also associated with a greater degree of pCO_2_ variability [4]. Preterm and unstable infants are more susceptible to changes in cerebral blood flow and display pressure passivity [5]. Furthermore, fluctuations of pCO_2_ can adversely affect cerebral oxygenation and perfusion and the electrical activity of the brain [6]; such fluctuation is predictive of adverse neurodevelopmental outcomes [7]. We have demonstrated that not only high EtCO_2_ levels during resuscitation in the delivery suite were associated with IVH development but also inflations resulting in larger tidal volumes and lower EtCO_2_ levels [3]. Those findings were subsequently confirmed by Mian and colleagues who found that tidal volumes in excess of 6 ml/kg were associated with cerebral damage in the neonatal period [8]. A further aim, therefore, of this study then was to test the hypothesis that infants exposed to the largest fluctuations in EtCO_2_ levels during resuscitation in the delivery suite would be at greatest risk of developing an IVH.

## Methods

We reviewed the records of infants born at less than or equal to 33 weeks of gestational age who required resuscitation in the delivery suite between March 2010 and March 2014. A certain part of their data was reported in a previous paper [3]. Ethical approval was given by the Outer London Ethics Committee for the data collection. The committee required parental consent only for analysis of the data, which was obtained once the mother was transferred to the postnatal ward. This study was approved by the King’s College Hospital NHS Foundation Trust Research and Audit Department.

### Resuscitation protocol

The resuscitation protocol, together with the monitoring methodology, has previously been described [3]. The clinicians attending the resuscitations had all been trained in newborn life support and had received the Resuscitation Council (UK) Newborn Life Support provider certificate. They had also been trained to operate the respiratory function monitor. During the resuscitation, the respiratory function monitor was set to display tidal volume, flow, inflation pressure and EtCO_2_ levels.

### Resuscitation monitoring

The resuscitation recordings were analysed to determine the duration of resuscitation, the number of inflations, the inflation pressure, the positive end expiratory pressure (PEEP), inflation time (Ti), mean airway pressure (MAP), expiratory tidal volume (V_TE_) of each inflation and the breath-by-breath minute ventilation. The highest EtCO_2_ was recorded as were the number of inflations with tidal volumes more than 6 mls/kg (high tidal volumes) or end-tidal CO_2_ levels of less than 4.5 kPa (low EtCO_2_ levels) and the number of inflations with both high tidal volumes and low EtCO_2_ levels as previously described [3]. The degree of fluctuation of EtCO_2_ (delta EtCO_2_) during resuscitation was determined by calculating the difference between the highest and lowest EtCO_2_ value during resuscitation in delivery suite.

### NICU monitoring

The unit’s routine policy was to insert arterial lines for blood gas sampling, and in the absence of such, access capillary sampling was undertaken. Blood gases were recorded on the intensive care observation charts every 4 to 6 h depending on the clinical status of the infant, and the hourly ventilator settings were collected at the time as each arterial gas result. Venous blood gases were not analysed in this study. Data on the maximum, minimum and average pCO_2_ value on each of the first, second and third days after birth were collected. The degree of fluctuation of CO_2_ (delta CO_2_) during each of the first 3 postnatal days was determined by calculating the difference between the highest and lowest CO_2_ value on each day. Volume targeted ventilation was not used on the NICU during the study period.

The medical records were examined to determine whether the infants developed an IVH which was diagnosed from cranial ultrasounds. As per unit policy, cranial ultrasounds were performed on admission to the unit for all infants born below 34 weeks of gestation. Cranial ultrasounds were repeated on day 3, between days 7 and 10, days 14 and 21 and days 28 and 30. All cranial ultrasounds were reported by a neonatal consultant. The IVHs were reported as grades I to IV using the Papile classification [9]. Severe IVH was diagnosed as a grade III or IV IVH. Other data extracted from the medical records included the infant’s gestational age, birth weight and Apgar score at 5 min, and whether the mother had received corticosteroids antenatally or had chorioamnionitis.

### Statistical analysis

The data were tested for normality using the Kolmogorov-Smirnov test and found to be non-normally distributed; hence, differences were assessed for statistical significance using the Mann-Whitney *U* test or the chi-square test as appropriate. As infants who develop IVH tend to be more immature, regression analyses were performed to determine if the results remained statistically significant after correcting for differences in gestational age. The strength of any relationships of EtCO_2_ values during resuscitation with CO_2_ levels during the first 3 days after birth were assessed by calculating Spearman correlation coefficients. The performance of factors identified as significantly related to IVH development from the regression analysis were assessed by receiver operator characteristic curve analysis and estimation of the corresponding area under the curve (AUC). Statistical analyses were performed using JMP statistical software (JMP 13.0.0, SAS Institute Inc., NC, USA) and SPSS software 25.0 (SPSS Inc., Chicago IL, USA).

## Results

Three hundred and seventy infants were born at less than or equal to 33 weeks of gestation within the study period. There were 150 infant recordings available for analysis, but 56 had recordings of tidal volume or EtCO_2_ traces which were unsuitable for analysis, a further 24 were excluded due to a large leak throughout and 12 excluded due to missing neonatal unit observation charts. The remaining 58 infants were included in this study and had a median gestational age of 27.3 (interquartile range (IQR) 24.9–29.0) weeks and a birth weight of 0.90 (0.72–1.21) kg. The infants who were and were not included in this study did not differ significantly with regard to birth weight (*p* = 0.893) or gestational age (*p* = 0.785). Fifty-three of the 58 infants were ventilated on day 1 after birth. Thirty infants developed no IVH with 28 infants developing an IVH. Seven infants developed a grade 1, eight infants grade 2, five infants grade 3 and the remaining eight infants a grade 4 IVH, that is 13 infants developed a severe IVH. All infants who developed an IVH did so in the first 3 days after birth. Eleven infants developed an IVH on day 1 (9 grade 1–2), nine on day 2 (5 grade 1–2) and eight on day 3 (1 grade 1–2). There were no significant differences in CO_2_ levels on the NICU and IVH development.

The highest EtCO_2_ during resuscitation in the no-IVH group was 8.0 (7.1–8.4) kPa and 10.0 (8.9–11.2) kPa in the IVH group; the difference remained statistically significant after adjusting for differences in gestational age between the two groups (*p* = 0.0002). The highest EtCO_2_ during resuscitation in the no/non-severe group IVH was 8.2 (7.4–9.9) kPa and 10.2 (8.6–11.1) kPa in the severe IVH group; the difference remained statistically significant after adjusting for differences in gestational age between the two groups (*p* = 0.037). There was no significant difference in the mean tidal volume during resuscitation between infants in the no/non severe IVH group and the severe IVH group (*p* = 0.621).

The lowest EtCO_2_ during resuscitation in the no-IVH group was 4.2 (3.2–5.2) kPa and 3.5 (3.0–4.5) kPa in the IVH group, but this was not statistically significant after adjusting for the difference in gestational age between the two groups (*p* = 0.11). The lowest EtCO_2_ during resuscitation in the no/non-severe IVH group was 4.0 (3.2–5.1) kPa and 3.1 (2.7–3.5) kPa in the severe IVH group; the difference remained significant after adjusting for the difference in gestational age between the two groups (*p* = 0.041).

There were weak correlations between the highest end-tidal CO_2_ on delivery suite and the highest pCO_2_ on the neonatal unit on days 2 (*r* = 0.274, *p* = 0.047) and 3 (*r* = 0.383, *p* = 0.005).

The delta EtCO_2_ during resuscitation was significantly different between the no/non-severe IVH (4.3 (3.1–5.6) kPa) and severe IVH groups (7.2 (5.9–7.6) kPa) (*p* = 0.003) and between infants with any IVH (6.2 (5.4–7.5) kPa) or no IVH (3.8 (2.7–4.3) kPa) (*p* < 0.001) after adjusting for differences in gestational age. The delta CO_2_ level during resuscitation weakly correlated with the delta CO_2_ level on the neonatal unit on days 2 (*r* = 0.385, *p* = 0.004) and 3 (*r* = 0.423, *p* = 0.002).

The receiver operator characteristic curve analysis demonstrated that in predicting any IVH, the maximum level of EtCO_2_ on the delivery suite had an AUC of 0.856 and the delta CO_2_ during resuscitation had an AUC of 0.940. A delta EtCO_2_ equal or greater than 4.9 kPa predicted any IVH development with 96.4% sensitivity and 83.3% specificity.

## Discussion

We have demonstrated that preterm infants who developed an IVH had a higher maximum level of EtCO_2_ and greater degree of fluctuation of EtCO_2_ levels during resuscitation. In addition, we highlight that the delta EtCO_2_ level in the delivery suite had both a high sensitivity and specificity in predicting IVH development. Importantly, we have shown that abnormal EtCO_2_ levels during resuscitation did not necessarily translate into abnormal levels on the neonatal unit. Our findings, then suggest that it is early abnormal carbon dioxide levels in preterm infants that are important in IVH development. Previously, initial post-delivery levels of pCO_2_ were found to be significantly higher in infants with cerebral haemorrhage than those without, but by 8 h after birth, those differences were no longer significant [10], which are consistent with our findings of the significant association of abnormal CO_2_ levels in the delivery suite, but not the neonatal unit, with IVH development. Some studies have suggested that sicker infants undergo more blood gas analysis than more stable infants, hence, allowing detection of a greater degree of fluctuation in levels of carbon dioxide [2]. We continuously monitored EtCO_2_ during resuscitation in all infants included in our cohort, and therefore, the outcomes relating to fluctuation of carbon dioxide are not influenced by the infant’s degree of sickness. We speculate that by the time infants are admitted to the neonatal unit, clinicians have greater access to routine monitoring and would readily re-check abnormal levels of pCO_2_ by blood gas analysis, hence, limiting the degree of fluctuation between extremes of values and thereby achieving tighter control. This would explain our findings that it is the delivery suite levels of CO_2_ that are more influential regarding IVH development and why the extreme levels of carbon dioxide seen during resuscitation do not translate to neonatal unit levels of CO_2_ during the subsequent 72 h. We did not use volume targeted ventilation during the study period and this would have likely reduced fluctuations in CO_2_ levels on the NICU.

As previously mentioned [3], we did find low EtCO_2_ levels that were associated with IVH development, but this was no longer significant after adjustment for gestational age. Nevertheless, we highlight fluctuations in EtCO_2_ that are highly predictive of IVH development. Hence, it is important to avoid both high and low levels of EtCO_2_ during resuscitation. Our results emphasize that respiratory function monitoring should be helpful in the labour suite. It has been previously shown that resuscitators were unable to accurately assess chest wall movement [11, 12], and use of respiratory function monitoring (RFM) can result in less mask leak and a lower rate of excessive tidal volumes [13]. We have, however, previously emphasized that resuscitators must have appropriate training so they can accurately interpret the results of such monitoring in real time [14]. Eye tracking technology has demonstrated that providers spend more time looking at the exhaled tidal volume waveform data than any other RFM parameter to guide positive pressure ventilation [15] suggesting further training is required on the importance of EtCO_2_ levels during resuscitation.

Our study has strengths and some limitations. This is the first study to evaluate EtCO_2_ levels and fluctuation of EtCO_2_ levels during resuscitation and how they correlated with both carbon dioxide levels in the first 3 days after birth and IVH development. We monitored EtCO_2_ levels during resuscitation, yet on the neonatal unit, we have analysed levels of CO_2_ from blood gas measurements. Previous studies, however, have found EtCO_2_ to be as accurate as arterial monitoring when trending carbon dioxide levels [16]. We did not, however, measure cerebral blood flow in our cohort and hence, cannot speculate on the mechanisms behind our findings.

In conclusion, a greater degree of fluctuation of EtCO_2_ levels in the delivery suite was associated with a significantly increased risk in the development of IVH. Those results emphasise the need for EtCO_2_ monitoring during resuscitation in the delivery suite.

## Abbreviations

CO_2_: Carbon dioxide
EtCO_2_: End-tidal carbon dioxide
FiO_2_: Inspired oxygen fraction
IQR: Interquartile range
IVH: Intraventricular haemorrhage
pCO_2_: Partial pressure of carbon dioxide
PEEP: Positive end expiratory pressure
RFM: Respiratory function monitoring
Vte: Expiratory tidal volume
